# Feasibility of a commercial smartphone application for dietary assessment in epidemiological research and comparison with 24-h dietary recalls

**DOI:** 10.1186/s12937-018-0315-4

**Published:** 2018-01-09

**Authors:** Gina L. Ambrosini, Miriam Hurworth, Roslyn Giglia, Gina Trapp, Penelope Strauss

**Affiliations:** 1 School of Population and Global Health M431, University of Western Australia, 35 Stirling Highway, Crawley, Perth, Western Australia 6009 Australia; 2 Telethon Kids Institute, University of Western Australia, Perth, Western Australia Australia

**Keywords:** Dietary assessment, Diet surveys, Mobile health, Reliability, Validity, Limits of agreement, Food record, 24-h dietary recall

## Abstract

**Background:**

Dietary assessment methods that can provide high quality data while limiting participant burden and resource requirements in epidemiological research are highly sought after and continue to evolve. The use of mobile phone technology in research has increased rapidly over the last decade and offers multiple advantages to the researcher over traditional data collection methods. This study tested the acceptability and relative validity of a commercial smart phone application (app) for use as an epidemiological dietary assessment tool, compared with a traditional dietary assessment method.

**Methods:**

Study participants completed a 4-d food diary using a modified version of the *Easy Diet Diary* app and two 24-h dietary recalls during the same week, for comparison. At the end of data collection, participants completed a questionnaire on their experience with both methods. Average proportions of energy from macronutrients and fibre, iron, and calcium densities from the app and 24-h recalls were compared after log transformation, by calculating mean agreement, limits of agreement (LOA), and Pearson’s correlations. The prevalence of dietary under-reporting was compared in each method using the Goldberg method.

**Results:**

A total of 50 adults (82% women) provided data for analysis (mean age, 31 y; mean BMI, 22.4 kg/m^2^; 14% overweight or obese). Participant feedback showed high levels of acceptance of the app; 83% preferred using the app to completing 24-h dietary recalls. The average difference in energy intake (mean agreement) between methods was 268 kJ/d. For all intakes except alcohol, the average difference between methods was not significantly different from zero. Most limits of agreement were within an acceptable range. The prevalence of dietary misreporting was similar in both methods.

**Conclusions:**

These findings demonstrate good feasibility for applying this commercially-developed smartphone app in epidemiological research.

**Electronic supplementary material:**

The online version of this article (10.1186/s12937-018-0315-4) contains supplementary material, which is available to authorized users.

## Background

Reliable dietary assessment tools are essential in nutrition epidemiology and for advancing understanding of population dietary intakes, diet quality, dietary determinants, as well as diet-disease relationships, in public health research. The challenges in obtaining valid dietary assessments have been detailed and debated extensively [1]. As such, choices about which dietary assessment tool to administer in epidemiological studies are based on trade-offs between the relative validity of different dietary assessment methods (in the absence of methods that can provide a ‘true’ measure of dietary intake), their associated burden on the respondent and researcher, and the resources required to administer them [2]. Large epidemiological studies often utilise food frequency questionnaires (FFQs), which are designed to be quick to administer, summarise intake over an extended period of time, and have been shown to be able to rank individuals according to their dietary intakes [2]. By comparison however, food diaries and 24-h dietary recalls are considered more precise than FFQs [3, 4] and can provide meal-specific details, such as how the food was sourced, where a meal was eaten, and time of meal; which are valued by public health researchers and policy makers interested in understanding the factors that shape dietary choices. Developing dietary assessment methods that meet these requirements while keeping participant burden and resource requirements low are therefore highly desirable.

Evidence of the acceptability, convenience, and preference for using smartphone technology for dietary assessments is growing [5–7]. In Australia, smartphone ownership rates are high. Market research conducted in 2017 estimated that smartphone ownership ranged from 99% of 18–29 year olds to 49% of 65+ year olds, with ownership rates similar in metropolitan and regional areas [8]. Thus, there is potential to test whether the use of smartphone applications (apps) can feasibly improve the quality of dietary assessments. Due to their portability and social acceptability, an app-based food diary may be more convenient and more likely to be completed at the time of eating than a paper-based diary, thereby reducing the error associated with recalling dietary intake when recording it later. In addition, mobile dietary assessments enable the rapid transfer of digital data between the research participant and researcher, and real-time communication for monitoring participant progress, potentially reducing participant burden and improving data quality.

Most currently available app-based dietary assessment tools do not differ substantially from conventional methods [9–11] i.e. food diaries and 24-h recalls, as they rely on some form of self report. While a small number of mobile dietary assessment tools that capture photographic images of meals are emerging, at present they remain labour intensive and require considerable manual interpretation and coding of images [7]. A limitation in all self-reported dietary assessments is dietary under-reporting, which can be conscious or unconscious, is more prevalent among adolescents and overweight respondents, and may seriously bias observed diet-disease relationships [12]. With high levels of personal attachment to smartphones and their ability to capture real-time data, unintentional under-reporting might be reduced with the use of mobile dietary assessments [5, 13] however, this requires testing.

This study sought to test the feasibility, acceptability, and relative validity (or inter-method reliability [14]) of a commercially-developed smart phone application for use as a dietary assessment tool in epidemiological research (4-d food diary), in comparison to a traditional dietary assessment method (two 24-h dietary recalls). We report user experience and preferences for each dietary assessment method. We hypothesised that total energy and other nutrient intakes from each method would be in moderate agreement and that the proportion of dietary under-reporters would be lower when respondents used the app to record their dietary intake.

## Methods

### Procedure

A convenience sample was recruited through networks across the University of Western Australia, the Telethon Kids Institute, and nearby sports facilities in Perth, Western Australia, via institution and student newsletters, a dedicated Facebook page, study flyers, and word-of-mouth. Recruitment occurred between February and November 2016 and was limited to Australian residents. To be eligible for the study, participants had to be at least 18 years of age, have their own iPhone for use in this study (with iOS operating system version 8 or later), and not be on a medically restricted diet. As the research team had a number of projects planned on nutrition during pregnancy, women with a low risk pregnancy, who were less than 32 weeks pregnant, were not currently experiencing symptoms of morning sickness, and met all other study eligibility criteria, were recruited through parent and child playgroups as well as the aforementioned networks.

A baseline questionnaire was used to screen for study eligibility and, if eligible, collect basic information including contact details, height, weight, pregnancy status (incl. trimester), lactation status, education level, and country of birth. To classify individual physical activity level the following evaluated question was included: “In the past week on how many days have you done a total of 30 minutes or more of physical activity, which was enough to raise your breathing rate? This includes sport, exercise, and brisk walking or cycling for recreation or to get to and from places, but excludes housework or physical activity that may be part of your job” [15, 16]. All questionnaires were administered online via the Qualtrics platform.

### Dietary assessments

#### Smartphone application

**Fig. 1:**
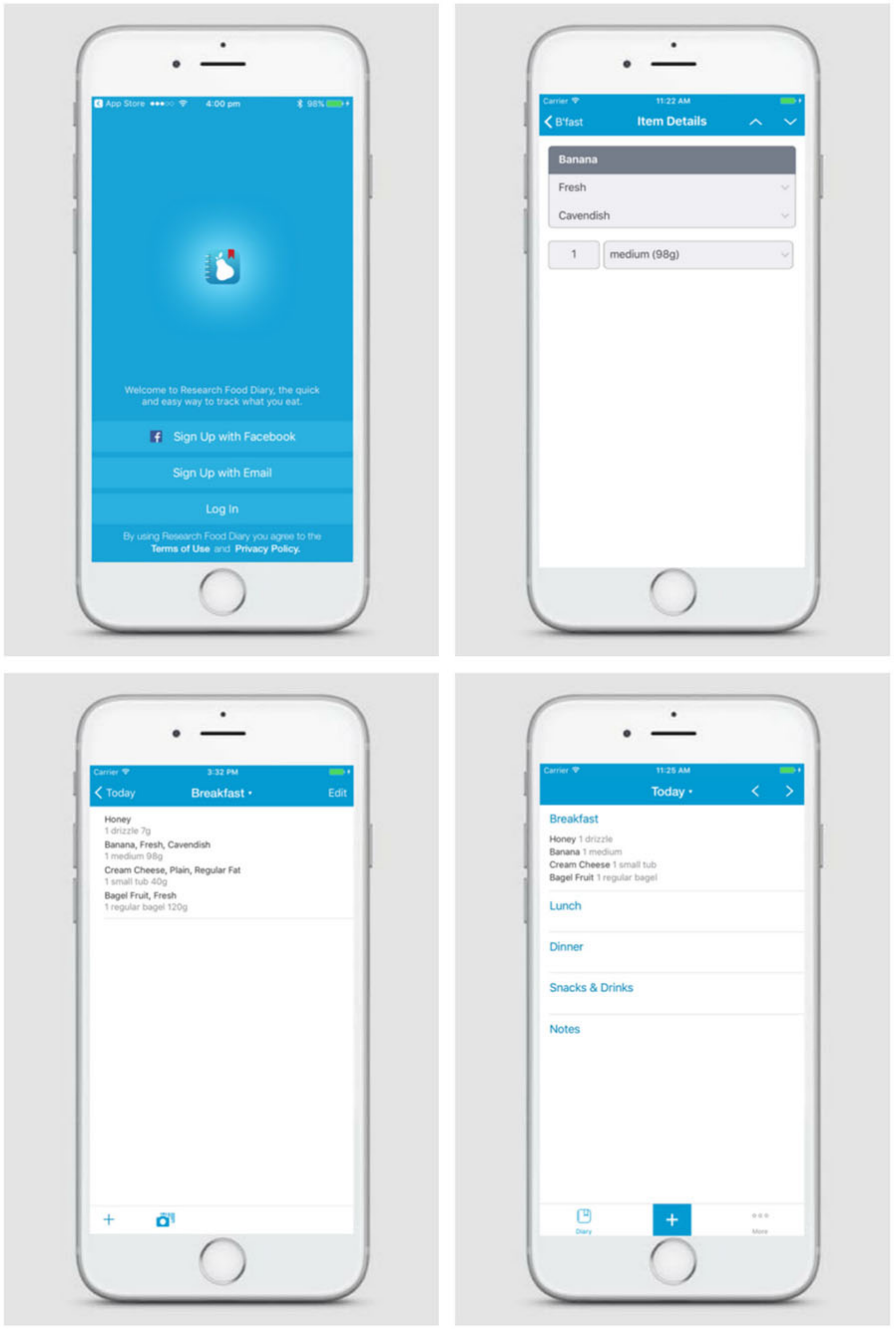
Screenshots of *Research Food Diary* (provided with permission from Xyris Software (Australia) Pty Ltd. [21])

The *Australian Calorie Counter - Easy Diet Diary* smartphone app is a commercial calorie counter, food diary, and activity tracker developed and owned by Xyris Software (Australia) Pty Ltd., first published as an iPhone app on iTunes in 2011 [17]. The *Easy Diet Diary* app was chosen for testing as a dietary assessment tool (i.e. food diary or record) in a research setting, for several reasons. It had already undergone significant development and testing, and had a high user rating (4.5 stars out of 5, based on 4753 ratings of all versions at the time of writing [17]). A strong feature of this app is its ability to allow the user to select their food or beverage (using a ‘search as you type’ interface (Fig. 1)) from a large database of ‘brand name’ commercial foods currently sold in Australia (*AusBrands 2015*) and a database of simplified food descriptors (*AusFoods 2015*). At present, the *Easy Diet Diary* is the only app for recording dietary intake that links with *AusBrands* and *AusFoods* (both databases developed by Xyris Software Australia Pty Ltd). Another feature is that the food diary can be emailed directly from the *Easy Diet Diary* app to the researcher, in a format that can be readily imported into *FoodWorks Professional* nutrient analysis software [18] and analysed using Australian food composition databases [19, 20]). The *Easy Diet Diary* app also supports scanning barcodes of many proprietary foods to record dietary intake.

As the aim of this study was to test a smartphone app for use in observational research, the *Easy Diet Diary* app was modified to remove all dietary feedback (e.g. calories consumed) that would usually be provided to the user, to minimise alterations in dietary intake during the recording period. The modified version of the *Easy Diet Diary* tested in this study is hereafter referred to as the *Research Food Diary* (RFD) app [21].

After providing informed consent and completing the baseline questionnaire, each participant was assigned to a study nutritionist, who telephoned them to answer any queries about the study and agree on a date to commence the 4-d food diary. Participants were sent instructions on how to download the RFD app and asked to record everything they ate and drank over four consecutive days, including one weekend day, using the app. As it was an aim of this study to assess the app’s ease of use and learnability, participants received information on the app’s basic functions, but no specific training was provided. For example, participants were asked to select the closest item description and portion size estimated to match their intake. Weighing of food was not required; the RFD app allows the user to enter portion sizes in household measures or using typical serve sizes.

After completing their 4-d food diary, participants emailed their diary to their study nutritionist, who checked it for completion and corrected any entries that had not imported properly. For example, any foods in the RFD app that did not have complete nutritional data in *FoodWorks* required substitution with an equivalent from the food composition database, to ensure recorded days’ data were not lost.

#### 24-h dietary recalls

To provide an estimate of usual dietary intake for comparison with the RFD 4-d diary, two 24-h dietary recalls were collected [22]. This comparison method was chosen to reduce correlated errors and inflated observed agreement between methods,[23] and is consistent with previous studies evaluating mobile phone dietary assessments [5, 24, 25]. Participants were contacted via telephone by their study nutritionist to conduct the 24-h dietary recalls unannounced. We aimed to collect at least one recall overlapping with the 4-d diet diary and one recall for a weekend day within 7 days of commencing the diet diary. This was to strike a balance between estimating dietary intakes over a short, comparable period of time, while limiting possible influence from respondents simply recalling their food diary for the same days (potentially inflating observed agreement). A structured interview based on the US Department of Agriculture’s multiple-pass telephone recall method was used [26]. The nutritionist entered each 24-h dietary recall into *FoodWorks Professional* software [18] for nutrient analysis.

### Participant feedback on dietary assessments

After completing both dietary assessments, participants completed an online questionnaire on their experiences using the app and undertaking recalls, such as ease of use, learnability, convenience and perceived accuracy. Responses were based on a 5-point Likert scale (strongly disagree, disagree, undecided, agree, or strongly agree with statements provided). Optional open-ended questions asked about perceived changes in dietary intake during recording and reasons for preferring either the app or recalls.

Participants were then sent feedback on their 4-d food diary. Feedback consisted of average daily proportion of energy (%) from all macronutrients and average daily micronutrient intake as a percentage of the Australian Recommended Dietary Intakes, appropriate for the participant’s reported age, gender, pregnancy or lactation status [27].

### Data cleaning

Nutrient analysis of the 24-h recalls and 4-d diaries was undertaken using *FoodWorks Professional* (v8) software [18] and Australian food composition databases [19, 20]. Supplementary nutrient intakes were not included. Implausible dietary intakes were identified as <3000 kJ/d or >20,000 kJ/d. Some recipes recorded using the RFD app required recoding due to a technical issue related to compatibility between the imported RFD app files and the nutrient analysis software. This had no effect on the recorded data except to make it usable.

### Statistical analyses

Mean (SD) daily intakes of total energy (MJ), the proportion of energy (%E) from macronutrients and for brevity, selected nutrient densities (chosen as indicators of diet quality (fibre g/MJ) or marginal population intakes (calcium mg/MJ; iron mg/MJ)) from each method were estimated for comparison. Participant characteristics in the baseline questionnaire were presented using descriptive statistics (means, standard deviations, proportions). Respondent feedback on each dietary assessment tool was assessed by summarising the frequencies and proportions of responses according to ‘agreement’ (strongly agree or agree) or ‘disagreement’ (‘strongly disagree or disagree) with statements in the questionnaire. Information provided by open-ended questions was described qualitatively. Characteristics of study completers and non-completers were compared using Fisher’s exact tests and independent t-tests.

Agreement between average daily nutrient intakes estimated by each dietary assessment was calculated using Bland and Altman’s methods [28, 29]. Bland-Altman graphs were used to: i) plot individual differences in intakes between methods (app - recall) against their mean (indicating whether the bias between methods was dependent on the magnitude of intake; this was formally tested by estimating the regression line of differences); ii) the mean agreement or bias between methods i.e. average of all differences between app and recall values (agreement at the group level); and iii) the limits of agreement (LOA), corresponding to two standard deviations either side of mean agreement, within which 95% of all individual differences lie. This approach helps identify the direction and consistency of any bias across intakes, and the potential for error (or differences) at the group and individual population level [29]. For some nutrients (%E from added sugar, polyunsaturated fat and alcohol; fibre density) the differences between methods were skewed; these variables were transformed to their natural logarithms (*ln*) to approximate normal distributions before analysis [28]. As many consumers reported zero intakes for alcohol, a constant value of 0.01 was added to all alcohol intakes to enable log transformation. To improve interpretation, mean agreement and LOA calculated using logged values were exponentiated and then converted to a percentage (multiplied by a factor of 100). This rendered agreement (or differences) as a multiple of the app’s estimate relative to that of the recall, with 100% indicating exact agreement. For example, mean agreement of 110% indicated that on average, the app’s estimate of average nutrient intake was 1.1 times that of the recall. All other variables showed normal distributions and are reported in their original units, to enable comparisons with other studies. Although correlations between nutrient intakes are a measure of association commonly reported in validation studies, they do not necessarily reflect agreement [30]. Pearson correlation coefficients (normally distributed data) and Spearman’s rank correlations (skewed data) were calculated to enable comparisons with other published studies.

The Goldberg cut-off method was used to identify dietary under-reporters in the app and 24-h recalls [31]. Participants were identified as dietary under-reporters if their ratio of average daily total energy intake (EI): basal metabolic rate (BMR) fell below an individualised cut-off. Participants with EI:BMR greater than their cut-off’s upper limit were classified as over-reporters. BMR was estimated by FoodWorks Professional software using Schofield equations incorporating gender, age, weight and height. Individual cut-offs for comparison with EI:BMR were estimated using the method recommended by Black [31]. This included assigning an individual physical activity level (PAL), which was based on the participant’s physical activity reported in their baseline questionnaire: sedentary (1.5), light (1.6), light-moderate (1.7), moderate (1.8), heavy (2.0) [18]. The statistical analyses were undertaken using STATA [32] and Microsoft Excel software.

## Results

A total of 87 adults volunteered to participate in this study. Of these, 18 were screened as ineligible or unable to participate: eight were on a medically restricted diet; six did not have an iPhone; four later declined for unknown reasons (Additional file 1: Figure S1).

Sixty-eight eligible adults provided written consent and completed the baseline questionnaire. Of these, ten were lost to follow up after multiple attempts to contact them to return their app data or complete 24-h recalls; four later refused to complete 24-h recalls; and four experienced technical issues e.g. could not update the required version of the iPhone operating system. The 16 non-pregnant participants who failed to complete the study after providing their consent had a higher average BMI (25.3 vs 22.3 kg/m^2^; *p* = 0.032) than non-pregnant completers, but did not differ significantly according to average age or distributions in education level, country of birth, gender, or pregnancy status (data not shown). The remaining 50 participants provided complete data for the present analysis.

Table 1 shows the main characteristics of the study population (*n =* 50). The average age was 31 years. The majority (82%) of participants were women, including eight (16%) women who were pregnant (two in trimester one; four in trimester two, two in trimester three). Average BMI (excluding pregnant women) was in the healthy weight range (22.4 kg/m^2^) and 14% of participants were overweight or obese. Most participants had completed university degrees (90%) and were born in Australia or New Zealand (64%).

**Table 1:** Participant characteristics

		n	*%*
Sex	Female	41	82
	Pregnant	8	16
	Non pregnant	33	66
	Male	9	18
Weight Status^a^	Underweight (BMI ≤18.5 kg/m^2^)	4	10
	Healthy (BMI 18.5–24.9 kg/m^2^)	32	76
	Overweight (BMI 25–29.9 kg/m^2^)	5	12
	Obese (BMI ≥ 30 kg/m^2^)	1	2
Highest Education Level Attained	University higher degree e.g. Masters or PhD	18	36
	University degree (undergraduate or Honours)	27	54
	Vocational e.g. TAFE course or certificate	4	8
	Year 12 secondary school	1	2
Country of Birth	Australia & New Zealand	32	64
	Overseas	18	36
		Mean	SD
Age (y)	Female	31.4	8.5
	Male	31.9	10.8
BMI (kg/m^2^)	All, non pregnant	22.3	2.7
	Female, non-pregnant	21.8	2.5
	Female, pregnant	26.7	6.2
	Male	24.9	2.4

^a^non-pregnant

### Between-method comparisons

Average daily nutrient intakes from the app and 24-h recalls are shown in Table 2.

**Table 2:** Mean daily nutrient intakes from each dietary assessment method (*n* = 50)

	Average of 4-d food diary (RFD app)		Average of two 24-h dietary recalls	
	Mean ± SD	Range	Mean ± SD	Range
Energy intake (kJ)	8854 ± 2267	4907, 16709	9123 ± 2337	4842, 14128
Protein intake (% E)	19.7 ± 4.9	12.1, 35.5	19.3 ± 4.3	10.2, 31.8
Total fat intake (% E)	34.0 ± 6.2	21.0, 50.1	34.8 ± 6.7	23.4, 57.8
Saturated fat (% E)	12.0 ± 2.8	4.6, 18.6	12.4 ± 2.9	4.2, 18.6
Polyunsaturated fat (% E)	6.0 ± 3.0	3.3, 22.3	6.0 ± 2.7	1.9, 20.5
Carbohydrate intake (% E)	39.8 ± 8.0	16.1, 56.5	40.4 ± 7.8	22.5, 57.6
Added sugar intake (% E)	5.0 ± 3.4	0.1, 15.0	6.2 ± 4.3	0.0, 16.5
Alcohol intake (% E)	2.1 ± 3.4	0.0, 15.2	1.3 ± 2.4	0.0, 9.5
Dietary fibre density (g/ MJ energy intake)	3.6 ± 1.3	1.9, 7.3	3.6 ± 1.2	2.0, 6.8
Calcium density (mg/ MJ energy intake)	117.6 ± 38.0	37.8, 295.5	111.7 ± 29.0	29.1, 187.6
Iron density (mg/ MJ energy intake)	1.6 ± 0.5	0.5, 2.9	1.5 ± 0.4	0.4, 2.8

**Fig. 2:**
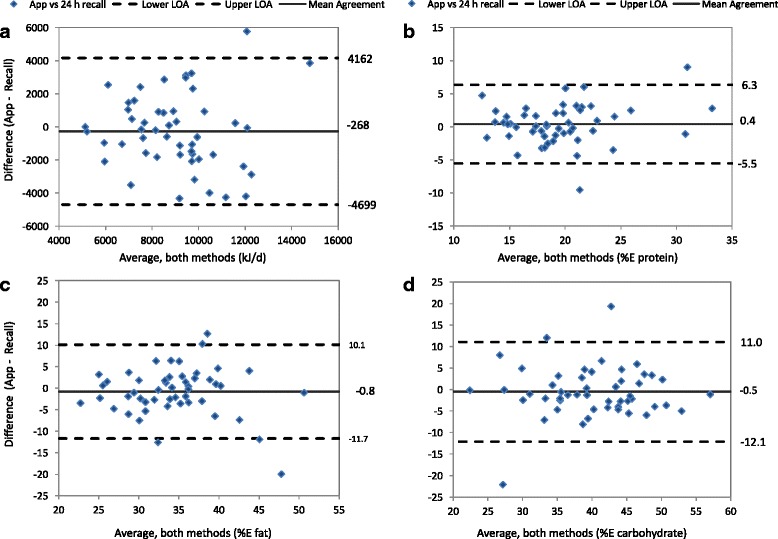
Bland Altman plots of individual differences between the app and 24-h recall estimates against their averages (diamonds); the upper and lower 95% limits of agreement (dashed lines); and mean agreement (solid line). **a** Total Energy Intake; **b** % Energy from Protein; **c** % Energy from Fat; **d** % Energy from Carbohydrate

Figure 2 shows Bland Altman plots of individual differences between the app and 24-h recall versus their means, the LOA, and mean agreement, for total energy intake and the major macronutrients. Mean agreement for total energy intake was −268 kJ/d (95% CI: -895, 358) and the limits of agreement were −4699 to 4162 kJ/d.

Mean agreement and limits of agreement between the app and recall for all nutrients examined are shown in Table 3. With the exception of alcohol, mean agreement (or differences) for all nutrients was not significantly different from zero, or 100% (logged data). Mean agreement between alcohol intakes (47%, 95% CI; 24, 92%) suggests that the app significantly underestimated alcohol intake in comparison to the 24-h recalls. The LOA indicated that individual differences between the app and recall were within acceptable ranges for protein, fat, saturated fat, carbohydrate, and iron densities (Table 3). However, there were larger individual differences for other nutrients, particularly added sugars (LOA:13–922%) and alcohol (LOA: 0.5–5266%), which showed the poorest agreement. Whereas, Pearson’s correlation coefficients for added sugar and alcohol were *r* = 0.68 (95% CI; 0.49, 0.80) and *r* = 0.65 (95% CI; 0.46, 0.79), respectively (Table 3). Other correlation coefficients ranged from *r* = 0.42 (iron density) to *r* = 0.79 (%E protein) (Table 3).

**Table 3:** Inter-method agreement, limits of agreement (LOA) and correlation coefficient (r) between RFD app and 24 h dietary recalls (*n* = 50)

	Mean agreement (95% CI)	LOA	Correlation Coefficient (95% CI)
Energy intake (kJ/d)	−268 (−895, 358)^a^	−4699, 4162^a^	0.52 (0.28, 0.70)
Protein (% E)	0.4 (−0.4, 1.2)^a^	−5.5, 6.3^a^	0.79 (0.66, 0.88)
Total fat (% E)	−0.8 (−2.3, 0.7)^a^	−11.7, 10.1^a^	0.63 (0.43, 0.77)
Carbohydrate (% E)	−0.5 (−2.2, 1.1)^a^	−12.1, 11.0^a^	0.72 (0.56, 0.83)
Saturated fat (% E)	0.5 (−0.2, 1.2)^a^	−4.6, 5.5^a^	0.60 (0.39, 0.76)
Calcium density (mg/MJ)	5.9 (−4.0, 15.8)^a^	−64.4, 76.1^a^	0.45 (0.20, 0.65)
Iron density (mg/MJ)	0.05 (−0.08, 0.18)^a^	−0.89, 0.98^a^	0.42 (0.16, 0.62)
Polyunsaturated fat (% E)	100 (92, 108)^b^	57, 174^b^	0.64 (0.44, 0.78)^c^
Added sugar (% E)	110 (78, 140)^b^	13, 922^b^	0.68 (0.49, 0.80)^c^
Alcohol (% E)	47 (24, 92)^b^	0.4, 5266^b^	0.65 (0.46, 0.79)^c^
Dietary fibre density (g/MJ)	100 (93, 107)^b^	60, 167^b^	0.66 (0.46, 0.79)^c^

^a^Mean and LOA agreement based on raw data (absolute differences, original units): 4-d diary average (app) - average of two 24-h recalls

^b^Mean agreement and LOA based on log transformed data (relative differences, %): 4-d diary average (app): average of two 24-h recalls

^c^Spearman’s rank correlation coefficient

For all nutrients except added sugars, individual differences between the app and 24-h recall estimates did not vary significantly by magnitude of intake. There was however, bias between methods for added sugars, which increased significantly as intakes of added sugars increased (beta = 0.36; *p* = 0.006).

### Dietary under-reporting

Individual estimated Goldberg cut-offs indicated that the prevalence of dietary under-reporting was 14% (*n* = 7) in the 24-h recalls and 16% (*n* = 8) in the RFD app. Three participants (6% of total) were classified as under-reporters by both dietary assessments. No participants were classified as dietary over-reporters.

### Participant feedback on the RFD app and 24-h recalls

Table 4 summarises participant feedback. The majority of respondents agreed or strongly agreed that: the app was easy to learn (94%); was easy (82%) and convenient (80%) to use; and the bar code scanner was a useful feature (77%). Most reported they were satisfied while using the app (80%) and preferred using the app (83%) to completing 24-h dietary recalls. The most popular reasons given for this preference included convenience, ease of use, perceived accuracy, being less time consuming, portable, and easy to remember.

**Table 4:** Respondent feedback on dietary assessments

Statement (n respondents)^a^	Agree/Strongly Agree n (%)	Undecided n (%)	Disagree/Strongly Disagree n (%)
Learning how to use the app was not difficult (*n* = 50)	47 (94)	1 (2)	2 (4)
The app was convenient to use (*n* = 49)	39 (80)	5 (10)	5 (10)
The foods I usually eat were easy to find on the app (*n* = 50)	26 (52)	8 (16)	16 (32)
I often recorded my food and drinks straight away (*n* = 50)	29 (58)	5 (10)	16 (32)
I found the app easy to use (*n* = 50)	41 (82)	6 (12)	3 (6)
The bar code scanner was useful (*n* = 43)	33 (77)	5 (12)	5 (12)
The app is likely to measure my diet accurately (*n* = 50)	37 (74)	7 (14)	6 (12)
I found it easy to estimate portion sizes (*n* = 50)	25 (50)	12 (24)	13 (26)
Overall I was satisfied using the app (*n* = 50)	40 (80)	4 (8)	6 (12)
I often included my own recipes when using the app (*n* = 42)	27 (64)	2 (5)	13 (31)
	App n (%)		Recall n (%)
Which method of dietary collection did you prefer? (*n* = 48)	40 (83)		8 (17)
Why did you prefer the app or recalls? (*n* = 40)^b^			
More convenient	29 (60)		2 (4)
Easier to complete	28 (58)		5 (10)
More accurate	24 (50)		5 (10)
Less time consuming	21 (44)		5 (10)
Easy to remember	20 (42)		1 (2)
Portable	20 (42)		–
Enjoyable to use	4 (8)		–
Learnt something new	3 (6)		–

^a^Questions were not compulsory therefore the total number of respondents varies by question

^b^Multiple responses were permitted

Nearly three-quarters (74%) of respondents reported they thought the app was a more accurate method than the recalls. Sixty four percent (64%) reported often inputting their own recipes when using the app, suggesting many were confident using the app. However, 50% reported finding it easy to estimate portion sizes with the app, 52% found foods that they usually eat easy to find on the app, and 58% reported recording their food straight away.

## Discussion

The results of this study indicate acceptable group level agreement between average daily nutrient intakes estimated by a food diary collected using a smartphone app and two 24-h dietary recalls. The average difference in total energy intake was acceptable (268 kJ/d) and there was little evidence of systematic bias between methods.

Interest in the use of mobile phone technology in health research is expanding rapidly and presents an opportunity to collect health data in ways that are less obtrusive and burdensome for the respondent and may even increase participant interest in research [33]. All of these factors may lead to better response rates, less study attrition, and could potentially support data collection periods over longer periods of time [34, 35]. Participants in this study indicated strong support for using the RFD app over completing 24-h recalls over the phone, citing convenience, ease of use and portability as key reasons, even without the provision of specific training on how to use the app and without immediate feedback from the app. This is consistent with similar studies which have shown a preference for smartphone technology to undertake dietary assessment when compared with traditional methods [6, 7].

A strength of the RFD app tested in this study is the large range of foods, branded and generic, available for user selection, owing to its interface with the continuously updated *AusFoods* and *AusBrands* databases, which are not currently available in other food diary recording apps. The RFD app includes a barcode scanner, which can assist the user to identify foods in Australian food composition databases, make recording quicker for the user, and possibly reduce food selection error. In addition, data from the RFD app is imported directly into *FoodWorks* nutrient analysis software, ready for data cleaning and analysis. This significantly reduces the time required for expert input, compared to administering 24 h dietary recalls.

We are aware of three other studies that have evaluated a mobile phone app linked to Australian food composition tables. Rangan and colleagues [24] compared energy and nutrient intakes from a 5-d food diary collected using the purposely designed “e-DIA” app, with those from three 24-h recalls, in 80 university students aged 19–24 years (62% female). Mean agreement was generally good; the average difference in total energy was 34 kJ (LOA −4062 kJ, 4130 kJ), no bias was seen between methods (differences did not vary by magnitude of intake) but some LOA were wide, indicating that the app provided energy and nutrient intakes that were in good agreement with a comparison method, at a group level.

Another Australian study evaluated estimated energy intake (EEI) based on a 4-d food diary recorded using a purposely designed, imaging-based, “FoodNow” app [35]. Participants were required to use the app to take two images of their meals with a fiducial marker, plus record a text description of the meal, including brand names and amounts of each food, before eating. Trained nutritionists then coded these images and text descriptions in duplicate. In a sample of 56 adults aged 18–30 y (mostly healthy weight women), EEI from the FoodNow 4-d diary was compared with measured energy expenditure (MEE) estimated using a SenseWear™ armband (multisensor monitor) worn for 7 days. Mean agreement between EEI and MEE was 826 kJ/d, and the LOA were −3709 kJ to 2057 kJ, with no bias indicated.

Another smartphone-based dietary assessment tool (“DietBytes”) that collects images and text descriptions of meals has been evaluated in an Australian setting. Intakes from a 3-d food diary collected on non-consecutive days using DietBytes were compared to three 24-h recalls collected on random days over three subsequent weeks [25]. The sample consisted of 25 pregnant women, average age 28.8 years, mostly in their 2nd trimester of pregnancy. The mean difference in energy intake between methods was 517 kJ/d. The LOA were similar to those in the present study and those reported by Rangan et al. [24], and there was no evidence of systematic bias.

One of the first studies to evaluate a smartphone app for dietary assessment was conducted in the UK, using the My Meal Mate (MMM) app, originally designed to assist with weight loss. The study included 50 adults (72% women) and compared a 7-d food diary using MMM against two 24-h dietary recalls [5]. The average difference in energy intake between methods was 218 kJ/d, with LOA ranging from −2434 kJ to 2022 kJ. The MMM app was also found to be useful for estimating group means of carbohydrate, fat and protein intakes.

We observed poor agreement for added sugars and alcohol in the present study. Sugar and alcohol intakes were found to be under-reported in the Australian study by Rangan et al. [24]. This may be due to selective under-reporting of foods containing added sugars (in the RFD app) or it may be that respondents reduced their alcohol or sugar intake whilst using the app. Given that alcohol intake is likely to be sensitive to day of the week, evaluated questionnaires designed specifically to measure alcohol intake may be more appropriate than a brief dietary assessment.

While only a small number of participants reported difficulties using the app in this study, a user interface that supports the easy location of foods in the database and selection of serving sizes are important design factors. This study sought to test the app with minimal intervention however, providing participants with additional technical information on how to use the app may further improve data quality.

The estimated prevalence of dietary under-reporting in this study was similar in the app and 24-h recalls. These results should be interpreted carefully, as when applied at the individual level, Goldberg cut-offs have lower sensitivity and specificity than assessing dietary misreporting at the group level [36]. However, including individual estimates of PAL can improve cut-off sensitivity [36]. In this study, individual PAL was based on physical activity level assessed using a single-item method which has been reported to perform as well or better than self-report questionnaires, when compared to accelerometer data [16]. Furthermore, the prevalence of dietary under-reporting in this group of predominantly healthy weight females is comparable with that reported in the latest Australian National Nutrition and Physical Activity Survey (ANNPAS 2011–2012), which was 16% for normal weight women (10% for normal weight men) [37].

We acknowledge other limitations in this study. The final sample included 50 adults, similar to the studies reviewed earlier that have evaluated mobile dietary assessment methods [5, 24, 35]. The sample size and the study population being predominantly healthy weight (14% overweight or obese vs 52% of 25–34 y in the latest National Health Survey 2014–15 [38]) may limit generalisability. A convenience sample was sought as reliability studies are time consuming and labour intensive for participants, making recruitment challenging. The source of recruitment for this study may have resulted in a more educated and motivated study population, not unlike the other reliability studies reviewed here [24, 35] and elsewhere [7]; this remains a challenge in this field and the results should be interpreted accordingly.

Twenty-four hour dietary recalls are frequently used in large population-based surveys e.g. US National Health and Nutrition Examination Survey. However, 24-h recalls are not error-free, nor a ‘gold standard’ reference method, and may produce errors similar to those in food diaries e.g. a portion size error, or failing to recall or record food that is eaten. An objective measure of energy expenditure is a preferred comparison method, but was beyond the scope of this study. However, the current study shows that a 4-d food diary collected using an app can provide nutrient estimates that are comparable (at a group level) with a conventional method (24-h recall) that requires considerably greater expert input and labour, and associated resources. This concurs with other studies that have found mobile phone dietary assessment methods perform similarly to conventional methods [6, 7]. Although smartphone ownership is common in Australia, [8] traditional data collection methods may still be required for a small proportion of participants without access to these devices. Finally, unlike other reliability studies [7] we applied the Bland-Altman method to evaluate agreement, rather than relying solely on correlation coefficients, which can give a misleading impression of agreement.

## Conclusions

These findings indicate that for population health research, the commercially developed RFD smartphone app is practical, can provide useful estimates of nutrient intake at the group level, and is acceptable to research participants. Further testing against an objectively measured reference method is recommended.

## Additional file

Additional file 1: CONSORT 2010 Flow Diagram. (DOCX 51 kb)

## Abbreviations

App: (smartphone) application
BMR: Basal metabolic rate
PAL: Physical activity level
RFD: Research Food Diary
