# Supplementary material for: Feasibility of a commercial smartphone application for dietary assessment in epidemiological research and comparison with 24-h dietary recalls
Source: Nutr J. 2018 Jan 9;17:5. doi: 10.1186/s12937-018-0315-4 (PMC5761106; doi:10.1186/s12937-018-0315-4)
Supplement: Additional file 1: — CONSORT 2010 Flow Diagram. (DOCX 51 kb) [file 12937_2018_315_MOESM1_ESM.docx]

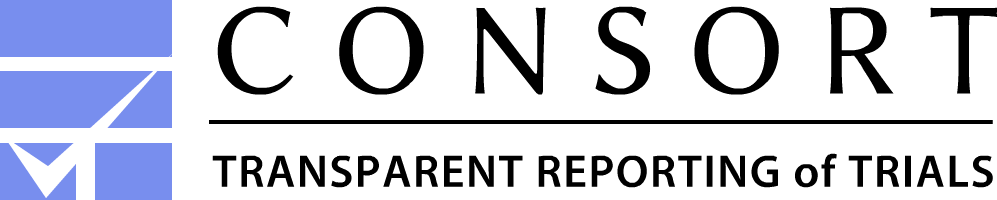


**CONSORT 2010 Flow Diagram**

Assessed for eligibility (n= 87)

S

## Enrolment

Analysed (pregnant n= 8, non-pregnant = 42)

Excluded (n= 18)

♦  Did not meet inclusion criteria (n= 14)

♦  Declined to participate (n=4)

Lost to follow-up as did not return contact (pregnant n= 1, non-pregnant n= 9)

Refused to undertake 24-hr recalls (non-pregnant = 4)

Discontinued study due to technical problems (pregnant n= 1, non-pregnant = 3)

## Follow-Up

Pregnant (n =10)

Non-Pregnant (n=58)

## Participant Type

## Analysis
